# Evidence of macrophagous teleosaurid crocodylomorphs in the Corallian Group (Oxfordian, Late Jurassic) of the UK

**DOI:** 10.7717/peerj.1497

**Published:** 2015-12-17

**Authors:** Davide Foffa, Mark T. Young, Stephen L. Brusatte

**Affiliations:** 1 School of GeoSciences, University of Edinburgh, Edinburgh, United Kingdom; 2 Ocean and Earth Science, University of Southampton, Southampton, United Kingdom

**Keywords:** Steneosaurus, Machimosaurus, Thalattosuchia, Corallian Gap, Teleosauridae

## Abstract

Teleosaurids were a group of semi-aquatic crocodylomorphs with a fossil record that spanned the Jurassic Period. In the UK, abundant specimens are known from the Oxford Clay Formation (OCF, Callovian to lower Oxfordian), but are very rare in the Kimmeridge Clay Formation (KCF, Kimmeridgian to lower Tithonian), despite their abundance in some contemporaneous deposits in continental Europe. Unfortunately, due to the paucity of material from the intermediate ‘Corallian Gap’ (middle to upper Oxfordian), we lack an understanding of how and why teleosaurid taxic abundance and diversity declined from the OCF to the KCF. The recognition of an incomplete teleosaurid lower jaw from the Corallian of Weymouth (Dorset, UK) begins to rectify this. The vertically oriented dentition, blunt tooth apices, intense enamel ornamentation that shifts to an anastomosed pattern apically, and deep reception pits on the dentary unambiguously demonstrates the affinity of this specimen with an unnamed sub-clade of macrophagous/durophagous teleosaurids (‘*Steneosaurus*’ *obtusidens* + *Machimosaurus*). The high symphyseal tooth count allows us to exclude the specimen from *M. hugii* and *M. mosae*, but in absence of more diagnostic material we cannot unambiguously assign DORCM G.3939 to a more specific level. Nevertheless, this specimen represents the first mandibular material referable to Teleosauridae from the poorly sampled middle-upper Oxfordian time-span in the UK.

## Introduction

Teleosauridae (Thalattosuchia: Crocodylomorpha) was a group of crocodylomorphs that inhabited lagoonal/coastal environments in the Jurassic (Andrews, 1909; Andrews, 1913; Buffetaut, 1982; Vignaud, 1995; Young et al., 2014a). During the late Middle and Late Jurassic a group of teleosaurids achieved large body sizes, heavily built skulls and blunt dentition indicative of a derived macrophagous feeding habit (Young et al., 2014a; Young et al., 2014b; Young et al., 2015a). This group includes *‘Steneosaurus’ obtusidens* from the Oxford Clay Formation of England (Callovian) and the genus *Machimosaurus* from the late Oxfordian to Tithonian of Europe and Africa (Sauvage, 1897–98; Hua et al., 1994; Lepage et al., 2008; Martin & Vincent, 2013; Young et al., 2014a; Young et al., 2014b; Young et al., 2015a; Martin, Vincent & Falconnet, 2015). It is worth noting that the presence of *Machimosaurus* in the upper Oxfordian is based on isolated tooth crowns from France and Portugal, and a partial lower jaw (Musée de la Princerie (Verdun, France), 2007.0.14) from France (Sauvage, 1897–98; Hua, 1996; Lepage et al., 2008; Young et al., 2014a; Young et al., 2014b). None of these specimens was found to be diagnostic at the specific level and they were referred to *Machimosaurus* sp. based on low tooth count (estimated as lower than *‘S.’ obtusidens*—see ‘Discussion’) and characters of the posterior mandible, which unfortunately cannot be assessed based upon the available *‘S.’ obtusidens* specimens.

The content of the genus *Machimosaurus* (sensu Young et al., 2014a) has been questioned by Martin and colleagues (2015). They consider *Ma. hugii* the only valid species within *Machimosaurus* (Martin & Vincent, 2013; Martin, Vincent & Falconnet, 2015). However, it should be noted that Martin and colleagues did not actually address the monospecifity within *Machimosaurus* as they focused on the validity of *Ma. buffetauti*. They suggest that this taxon is the same as *Ma. mosae*, and both should be referred to *Ma. hugii* as originally proposed by Martin & Vincent (2013). Their argument for the synonymy between *Ma. buffetauti* and *Ma. mosae* is based on the fact that the diagnoses produced by Young et al. (2014a) and Young et al. (2015b) to distinguish these two taxa would be either accountable for intra-specific variation or by post-mortem deformation on the specimens. We recognise that the specimens have undergone some deformation (as is the norm for fossil specimens); however, we disagree with Martin and colleagues conclusions and consider these differences true morphological traits—perhaps due to differing perception of morphological species. However, *Machimosaurus* species (*sensu*Young et al., 2014a) still differ from each other in stratigraphic occurrence, basioccipital apophysis cross-section, relative size and shape of the basioccipital tuberosities, relative size of the paroccipital processes and the expansion at their lateral ends, tooth morphology, tooth enamel surface morphology, and tooth count (exceeding modern crocodylian intra-specific variation Larson, 2013; Brown et al., 2015)). Perhaps more importantly, it should also be noted that the supposed synonymy of *Ma. buffetauti* and *Ma. mosae* does not prove the monospecificity of the genus *Machimosaurus*. Whilst Martin and colleagues appeal to the arguments proposed in Martin & Vincent (2013), the updated diagnosis that Young et al. (2014a) and Young et al. (2015b) proposed for *Ma. hugii* type species, and the unique tooth enamel morphology of the species (Young et al., 2014b), was not taken into account in Martin, Vincent & Falconnet (2015). Consequently, we do not find Martin, Vincent & Falconnet (2015) arguments to be compelling; as such we assert the taxonomic content of *Machimosaurus* as described in Young et al. (2014a) and Young et al. (2015b) to be valid and adopted it as phylogenetic framework in this paper.

Nevertheless, after a long debate, recent studies agree in considering *Machimosaurus* and *‘Steneosaurus’ obtusidens* morphologically distinct taxa (Martin & Vincent, 2013; Young et al., 2014a; Young et al., 2014b; Young et al., 2015a), which we agree with. Working under this assumption means that there is a significant gap separating *‘Steneosaurus’ obtusidens* and the first unambiguous *Machimosaurus* species. This time-span roughly corresponds with the Oxfordian stage, which in the UK is represented by a series of formations stacked in between the fossil-rich Oxford Clay Formation (OCF) and the Kimmeridge Clay Formation (KCF) (Cope, 2006). The middle-late part of the British Oxfordian has been referred to as ‘Corallian Gap’ due to the rarity of fossil vertebrates known from this period (Young, 2014). While little fossil material is known from this interval, the ‘Corallian Gap’ is significant because it marks a period of drastic changes in the sub-Boreal marine faunas. Marine reptiles were particularly affected, and plesiosaur, thalattosuchian and ichthyosaur fossils are rare in the Oxfordian and Corallian beds (Benton & Spencer, 1995) after being much more common in the Callovian beds. Additionally, the OCF marine reptile faunas are very different from those of the KCF in terms of both composition and taxonomy (Young, 2014). Unfortunately, the absence of diagnostic Corallian material hinders our understanding of how and why marine reptile faunas changed so dramatically during this time.

Teleosaurids are no exception to this pattern, as only rare, fragmentary, and mostly non-diagnostic material has been reported from the Oxfordian of the UK (Benton & Spencer, 1995). In contrast to other marine reptile groups, teleosaurids are, in fact, very poorly represented (only two genera) even in the fossil-rich Kimmeridge Clay Formation (Young & Steel, 2014; Young et al., 2014a). The *‘Steneosaurus’ obtusidens* + *Machimosaurus* sub-clade (Young et al., 2012; Martin & Vincent, 2013; Young, 2014)—common in continental Europe formations—is exclusively represented by a handful of teeth in the KCF (Krebs, 1967; Hua et al., 1994; Ruiz-Omeñaca, Piñuela & Garcia-Ramos, 2011; Martin & Vincent, 2013; Young & Steel, 2014; Young et al., 2014a). The paucity of teleosaurid material in the UK is puzzling. It may be a consequence of environmental changes (i.e., the UK becoming a deeper water environment), but this is still a matter of debate that can only be clarified by new discoveries and environmental-diversity studies.

**Figure 1:**
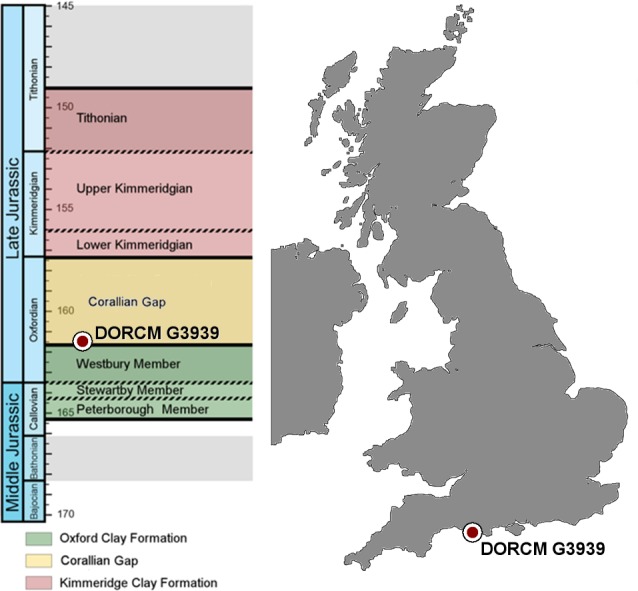
Stratigraphic chart and map. Middle-Late Jurassic stratigraphic chart and map of the UK. The red circles show the stratigraphic level and locality where DORCM G.3939 was found.

**Figure 2:**
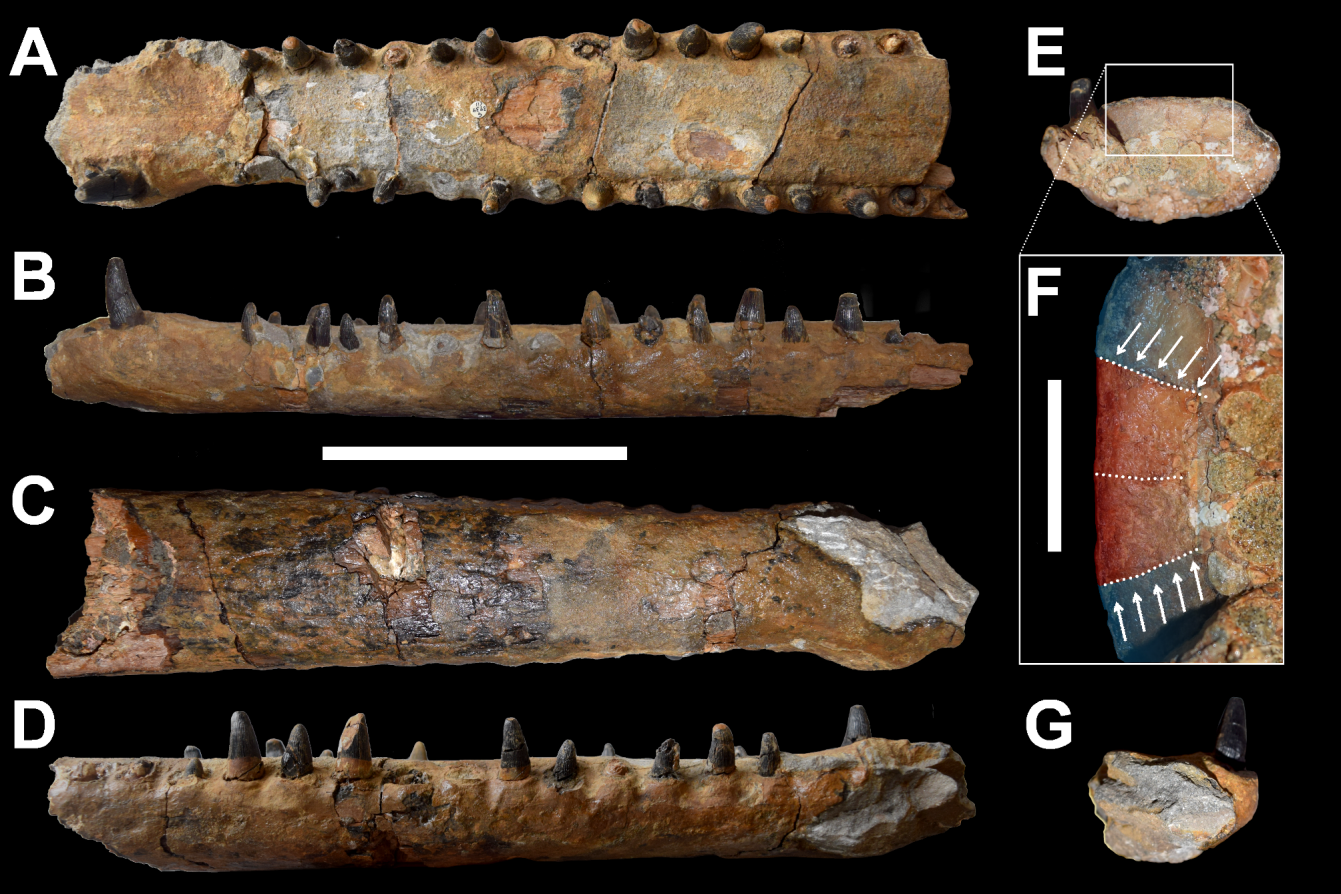
DORCM G.3939, *‘*Steneosaurus’ cf. *obtusidens*. Anterior mandibular symphysis in (A) dorsal, (B) left lateral, (C) ventral, (D) right lateral, (E) posterior, (F) close up of the posterior view, (G) anterior view. Dashed lines in (F) follow the sutures and arrows indicate the splenial-dentary sutures. Splenials are highlighted in red, dentaries in blue. Scale bars equal: 10 cm in (A–E) and (G); 2 cm in (F).

Here we describe a fragmentary but informative teleosaurid specimen (DORCM G.3939) from the Corallian of Weymouth Bay (Dorset) (Figs. 1 and 2). We demonstrate that it belongs to the sub-clade *‘Steneosaurus’ obtusidens* + *Machimosaurus* and that it shows close affinities to *‘Steneosaurus’ obtusidens*. However, as the genus ‘*Steneosaurus’* is still in need of revision, we are unable to recognize any unambiguous diagnostic features of a particular species, so we refer the specimen to *‘Steneosaurus’* cf. *obtusidens*.

## Background Information

The specimen, DORCM G.3939, was found at the Nothe Grit (SY30 686 787)—corresponding to the south flank of the Nothe Gardens by “S Winch Esq.” Paul Ensom (assistant Curator at DORCM at the time) physically collected DORCM G.3939, which was then presented to the Dorset County Museum (DORCM) in 1980, where it is currently on display in the Jurassic Gallery.

The location where the specimen was found is in close to the type section of the Nothe Grit, which is the base bed of the Corallian Group in the area (House, 1993). The Nothe Grit Member is a localised geological succession dated to the Oxfordian in South Dorset. It overlies the Oxford Clay Formation and constitutes a succession of thick beds of grey-yellow-brown sandstones, often including clay and extensive calcareous concretion (Coe, 1995). It is thought to represent a near-shore subtidal environment (House, 1993).

The specimen has never previously been formally described, and is not the *Teleosaurus* specimen from the same locality that is mentioned in Benton & Spencer (1995). In their review of the British Late Jurassic reptile sites, the authors briefly mentioned a crocodilian from “*Nothe, Weymouth (688788;* Teleosaurus *from Lower Calcareous Grit)*” (*sic* in Benton & Spencer, 1995). This specimen is not DORCM G.3939, as the authors were referring to a lower jaw erroneously referred to *Teleosaurus* (the specimen being pliosaurid) by Newton (1878), and later mentioned by Delair (1958, p. 60).

## Systematic Palaeontology

## Description and Comparisons

DORCM G.3939 is an almost complete and undistorted lower jaw that preserves the symphyseal region, and is approximately ∼30 cm long (Fig. 2). The anterior tips of both dentaries are incomplete so that the dentary alveoli 1 and 2 (D1, D2) are partially or entirely missing. The rest of the tooth rows continue up to the D19 alveolus on the right side and the D20 alveolus on the left side. Exceptionally, most of the teeth are intact and *in situ*, and two additional crowns were found associated with the specimen.

The dentary constitutes the vast majority of the symphyseal region as in all teleosaurids (Andrews, 1909; Andrews, 1913; Hua, 1999; Young et al., 2014a). The dentary is laterally expanded adjacent to the D3–D4 alveoli, resulting in these tooth crowns being located more laterally than the rest of the tooth row (Fig. 2A). The transverse plane of the D3–D4 alveoli is dorsal to the rest of the tooth row (Figs. 2B and 2D). Such a morphology is not unique in teleosaurids and also occurs in *Machimosaurus* spp. (MPV V1600bo, SMNS 91415), *‘Steneosaurus’ obtusidens* (NHMUK PV R 3168), and *S. edwardsi* (PETMG R178), and is present, although seemingly less pronounced, in other Callovian and Kimmeridgian longirostrine teleosaurids (e.g., Andrews, 1913; Lepage et al., 2008; Young et al., 2014a).

The D3 and D4 alveoli form a couplet, as they are separated only by a thin alveolar lamina, as in other teleosaurids (e.g., Andrews, 1913; Hua, 1999; Lepage et al., 2008; Martin & Vincent, 2013; Young et al., 2014a). Posterior to the D4 alveolus the interalveolar spaces are consistently large, but always shorter than the alveoli adjacent to each of them. These interalveolar spaces are comparatively shorter than in the longirostrine *Steneosaurus leedsi* (NHMUK PV R 3806), and similar to *S. edwardsi* (PETMG R175, PETMG R178), *S. herberti* (OUMNH J1420), *‘S.’ obtusidens* (NHMUK PV R 3168) and *Machimosaurus* spp. (Andrews, 1909; Andrews, 1913; Young et al., 2014a).

**Figure 3:**
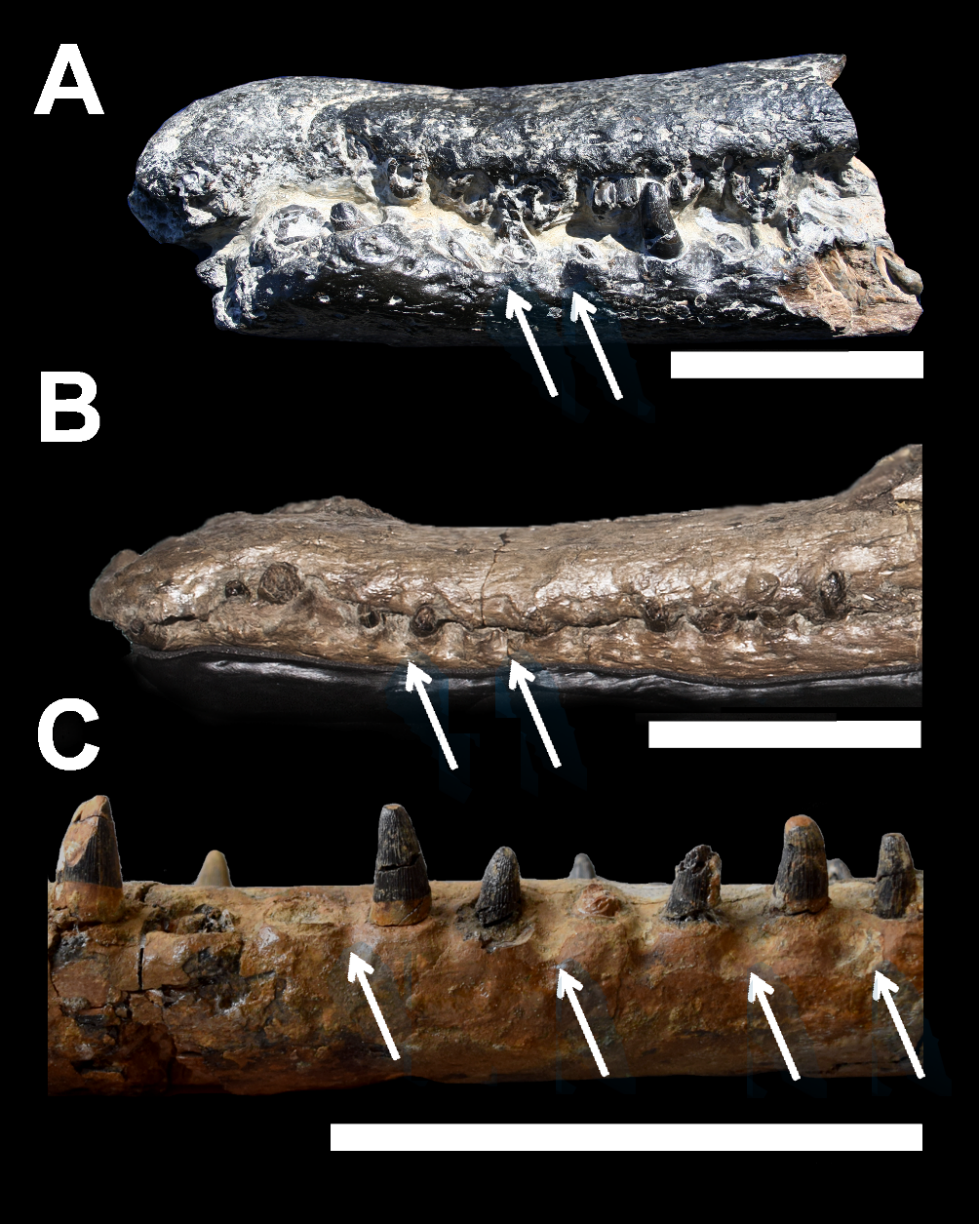
Comparative plate of macrophagous teleosaurid dentary pit-receptions. (A) *Machimosaurus buffetauti*, MPV V1600bo, left lateral view; (B) *‘Steneosaurus’ obtusidens*, NHMUK PV R 3168, left lateral view; (C) *‘Steneosaurus’* cf. *obtusidens*, DORCM G.3939, right lateral view. Arrows indicate reception pits for maxillary teeth. Scale bars equal 10 cm.

The interalveolar diastema between the D2 and D3 alveoli is hypothesised to have accommodated enlarged caniniform teeth from the premaxillae. Similarly the lateral surface of DORCM G.3939 dentary is deeply excavated between the alveoli to host opposing maxillary teeth (Fig. 3). Deep dentary ‘reception pits’ are not uncommon in Thalattosuchia and can be seen—not exclusively—in several macrophagous taxa (Andrews, 1913; Martin & Vincent, 2013; Young et al., 2014a; Young et al., 2014b). Within Teleosauridae, *‘Steneosaurus’ obtusidens* (NHMUK PV R 3168), *S. edwardsi* (PETMG R175), *S*. *herberti* (OUMNH J1420) and all known *Machimosaurus* species (Young et al., 2014a) display deep reception pits. These are also visible but considerably less pronounced in *Steneosaurus edwardsi* (PETMG R175), although this may be due to the poor preservation of the dentaries in this specimen.

**Table 1:** Comparative mandibular tooth count of Callovian-Kimmeridgian teleosaurids from UK formations. Data derive from available literature (Andrews, 1913; Young et al., 2014a) and direct observations on the specimens.

	Dentary tooth count	Dentary teeth adjacent mandibular symphysis	Dentary teeth anterior to the splenial	Symphyseal teeth along splenial
*Steneosaurus leedsi* (NHMUK PV R 3320)	43–44	33	24	9
? *Mycterosuchus nasutus* (CAMSM J1420)	40–41	36–37	21–22	15
*Steneosaurus edwardsi* (PETMG R175)	>28	26	–	–
*Steneosaurus herberti* (OUMNH J1420)	30	24	15	9
*Steneosaurus edwardsi* (PETMG R178)	>25	–	–	–
*‘Steneosaurus’ obtusidens* (NHMUK PV R 3168)	29	22–24	–	–
DORCM G.3939	>19–20 (28e)	23–24e	15–16e	6–7e
*Machimosaurus* sp. (Musée de la Princerie 2007.0.14)	>14 (24e)	>10	–	7–8
*Machimosaurus buffetauti* (SMNS 91315)	21–25	19–20	13	6–7
*Machimosaurus hugii* (NMS 7021)	>15	>13	11	>2
*Machimosaurus mosae*(Hua, 1999; Young et al., 2014a)	19	16	11	5

**Notes.**

e Estimate

The surface details (sutures and ornamentation) are largely obscured by the poor superficial preservation of the specimen (Figs. 2A–2D). The bone texture is not smooth but appears sculptured by deep anastomosed grooves similar to other large bodied macrophagous teleosaurid dentaries (Martin & Vincent, 2013; Young et al., 2014a).

The sutures of DORCM G.3939 are difficult to discern, although close examination of the posterior breakage surface reveals the contacts between the splenials and dentary laterally, and between the splenials along the midline (Figs. 2E and 2F). The splenial-dentary sutures are very close to the midline contacts, indicating that the mandible broke across the splenial anterior process. This can be assessed by looking at the lateral extent of the splenials, which in the posterior-most section of teleosaurid mandibular symphyses make up a considerable part of the entire mandible width. This contribution gradually decreases as the splenials taper anteriorly. When these observation are applied to DORCM G.3939, we can confidently conclude that in this specimen the splenial must have reached at least a few (estimated 3 or 4) alveoli anterior to the D19 alveolus. Unfortunately we cannot be sure of the exact extent of the splenial due to the poor visibility of the sutures in dorsal view. This estimate differs from all *Machimosaurus* specimens, where the splenial reaches up to the D13 alveolus in *Ma. buffetauti*, and the D11 alveolus in *Ma. mosae* (Hua, 1999; Young et al., 2014a) (Table 1). Unfortunately, comparisons cannot be extended to *Steneosaurus edwardsi* (PETMG R178) and *‘S.’ obtusidens* (NHMUK PV R 3168) because the dorsal surface of the lower jaws is inaccessible in both these specimens (Andrews, 1913; Table 2 in Young et al., 2014a) (Table 1).

The coronoids are not visible on either side, even in cross-section, demonstrating once again that the anterior break of the specimen occurred in front of the anterior-most extent of the coronoids. The importance of this observation is further elaborated upon in the Discussion.

**Figure 4:**
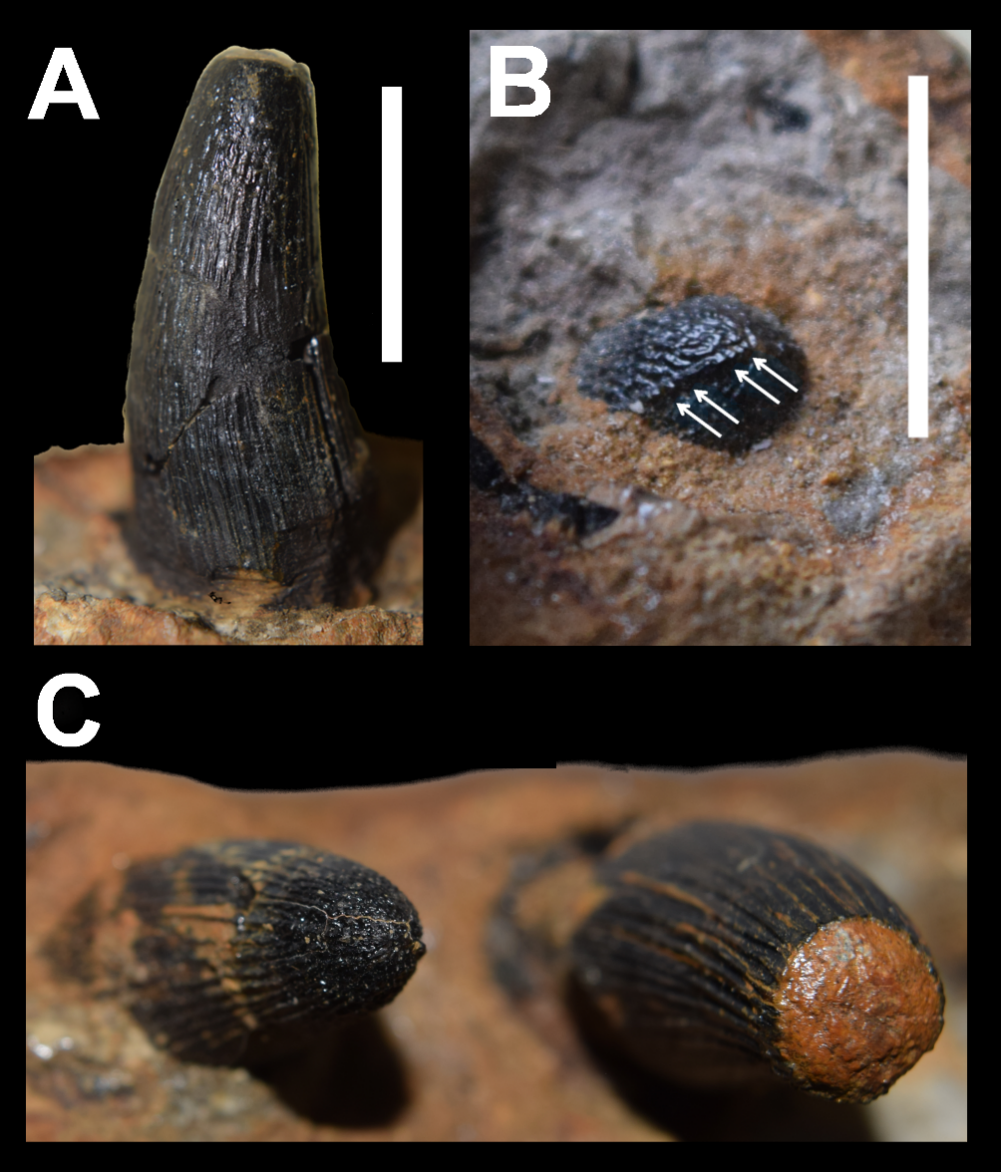
Dentition of DORCM G.3939, *‘Steneosaurus’* cf. *obtusidens*. (A) left D4 in labial/medial view; (B) left D3, showing details of the carina, blunt apex, anastomised ornamentation and denticles; (C) dorsal view of left D16 (right) and D17 (left). Scale bar equals 1 cm in (A); 0.5 cm in (B).

**Figure 5:**
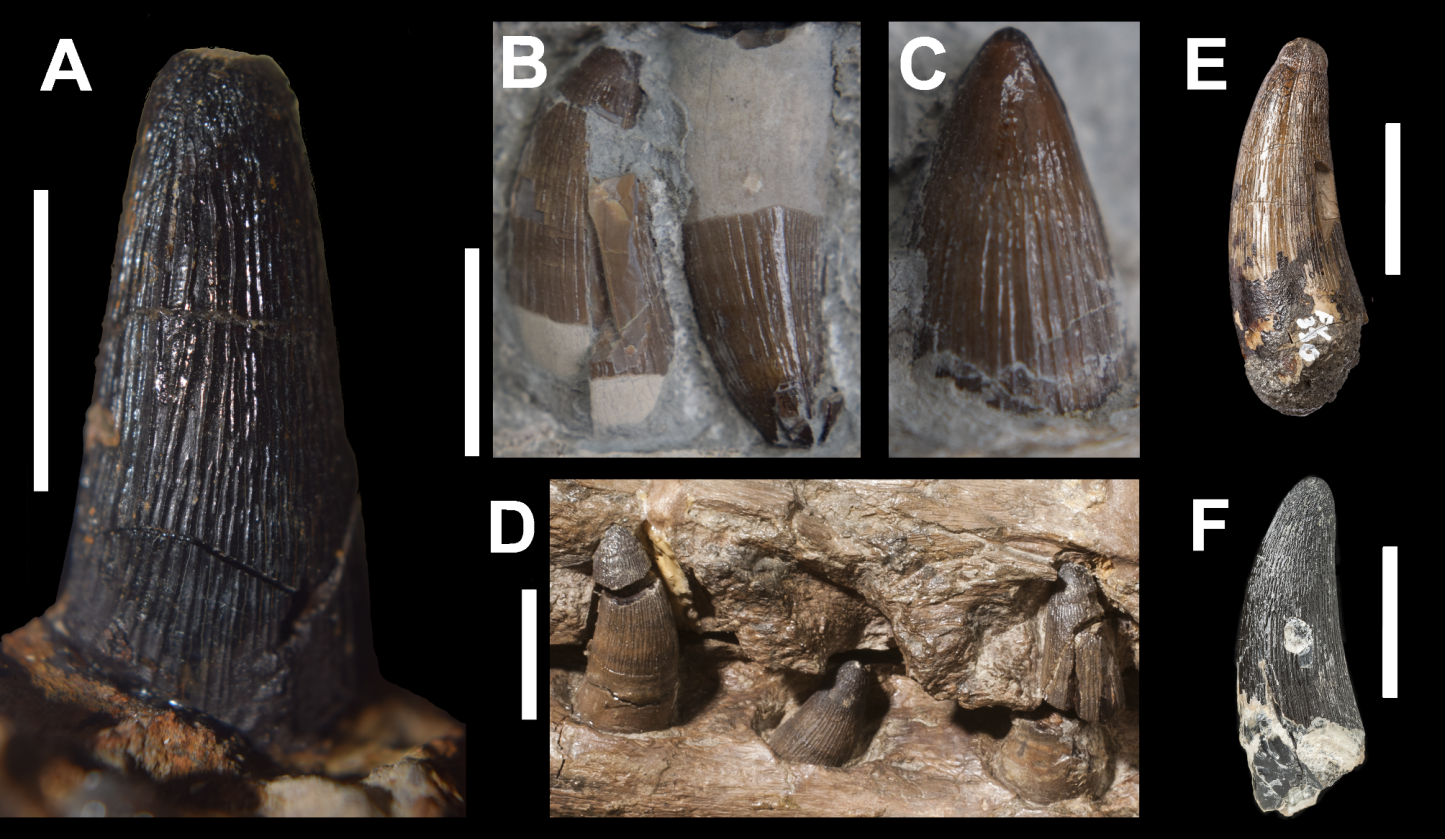
Comparative plate of teleosaurid teeth. (A) *‘Steneosaurus’* cf. *obtusidens*, DORCM G.3939, (B) *‘Steneosaurus’ edwardsi*, PETMG R178, referred specimen; (C) *‘Steneosaurus’ edwardsi*, PETMG R178, referred specimen, detail of growing dentary tooth; (D) *‘Steneosaurus’ obtusidens*, NHMUK PV R 3168; (E) *Machimosaurus buffetauti* DFMMh FV 330; (F) *Machimosaurus hugii*, NHMUK PV R 5, referred specimen. Scale bar equals: 1 cm in (A); 2 cm in (B), (D), (E) and (F); (C) not to scale.

The dentition of DORCM G.3939 is well preserved and virtually indistinguishable in shape, ornamentation and carinae from the dentitions of *‘Steneosaurus’ obtusidens* and *Machimosaurus* (Figs. 2, 4 and 5A). In DORCM G.3939 most of the preserved teeth are intact, and in life position are placed in vertically-oriented alveoli. The left D4 tooth is the largest preserved tooth crown, typical for teleosaurid jaws (Figs. 2, 4 and 5A) (Andrews, 1913). All the crowns are conical with a circular/sub-circular cross section. They are readily differentiated from the gracile teeth of longirostrine teleosaurids, such as *Steneosaurus leedsi* (NHMUK PV R 3320) and *Mycterosuchus nasutus* (NHMUK PV R 2617, CAMSM J1420 referred specimen). The teeth of DORCM G.3939 are robust, only slightly curved lingually and have a consistently small crown height-length ratio (H:L), spanning from 1.64 (right D13) to 1.95 (left D4). Whilst the teeth of *Steneosaurus edwardsi* (PETMG R175, PETMG R178), and *S. herberti* (OUMNH J1420) are comparatively more robust than those of *S. leedsi* and *My. nasutus*, they lack the distinctive surface enamel ornamentation that DORCM G.3939 shares with *‘S.’ obtusidens* and *Machimosaurus* spp. (Fig. 5). Every DORCM G.3939 tooth is consistently ornamented by parallel, densely packed, high-relief apicobasal ridges that are variable in length (Figs. 4A and 5A). This pattern is maintained up to 3/4 of the crown height, where the texture becomes anastomosed (Figs. 4A and 5A) (Young et al., 2014a; Young et al., 2014b; Young et al., 2015a). All teeth are distinctively carinated and have denticles that are non-contiguous and poorly detectable without visual aids (Young et al., 2015a); both features are clearly visible only close to the apex (Fig. 4B). Conversely, the presence of carinae is variable in *Machimosaurus* spp. The crowns of DORCM G.3939 display false and true zyphodonty as shown in ‘*Steneosaurus*’ *obtusidens* (Young et al., 2015a).

The unusually well preserved dentition and lack of distortion in the specimen shows that teeth proceeded in an alternating pattern (Figs. 2A, 2C and 4C). Fully grown teeth alternate with smaller crowns, which are likely representative of earlier developmental stages. This alternating pattern has been observed in modern reptiles before, and may be related to tooth replacement rhythm (Edmund, 1969). It would, in fact, guarantee that there is a fully grown crown every two tooth positions, minimising the disadvantage of continuous tooth replacement through life (Edmund, 1969). All the largest tooth apices are worn and polished (Fig. 4C). However, the apices of the smaller crowns are rounded as in *Machimosaurus* and *‘Steneosaurus’ obtusidens* and not pointed as in all other teleosaurids (Figs. 4B and 5) (Andrews, 1913; Lepage et al., 2008; Martin & Vincent, 2013).

## Discussion

The rarity of Oxfordian vertebrate fossils means that DORCM G.3939 is a pivotal specimen in understanding the evolution of teleosaurids. This specimen is particularly important because it fills a stratigraphic gap between *‘Steneosaurus’ obtusidens* and the species of *Machimosaurus*, and—excluding teeth—it is the youngest macrophagous teleosaurid yet known in the UK. The dentition of DORCM G.3939 unambiguously demonstrates that it belongs to the *‘Steneosaurus’ obtusidens* + *Machimosaurus* sub-clade of macrophagous teleosaurids. This confirms that the macrophagous teleosaurid lineage was still present in the UK at the beginning of the Corallian. This has immediate consequences because neither *‘Steneosaurus’ obtusidens* nor *Machimosaurus* has been reported from the early Oxfordian. This means that if DORCM G.3939 belongs to *Machimosaurus*, then the first appearance of this genus should be stretched to the very base of Oxfordian. Conversely, if DORCM G.3939 belongs to *‘Steneosaurus’ obtusidens*, then this specimen represents the latest occurrence of this taxon, and the first in the Late Jurassic.

Unfortunately, with the exception of the distinctive *Machimosaurus hugii*, *‘Steneosaurus’ obtusidens* and *Machimosaurus* teeth are virtually indistinguishable (Young et al., 2014a; Young et al., 2014b; Young et al., 2015a), so tooth morphology is insufficient to diagnose DORCM G.3939 at the specific level. However, although DORCM G.3939 is an incomplete jaw and has no unambiguous autapomorphies, what is preserved provides useful information that can help pin down its systematic affinities. The mandibular morphology is enough to demonstrate that DORCM G.3939 is more similar to ‘*Steneosaurus’ obtusidens* than to *Machimosaurus buffetauti*. This is based on mandibular symphysis tooth count and morphological features of the mandible.

First, DORCM G.3939 has a high tooth count. The DORCM G.3939 mandibular symphysis is incomplete, and thus the tooth count of 19–20 is a conservative estimate that is likely to be higher. We estimated ∼28 dentary teeth for this specimen based on the splenial and post symphyseal tooth count in *M. buffetauti* (SMNS 91315) and *Machimosaurus* sp. (Musée de la Princerie 2007.0.14) (Table 1). Importantly, macrophagous teleosaurids show a trend of decreasing snout length and tooth count. This trend starts in the Callovian *‘Steneosaurus’ obtusidens* (∼29 dentary teeth; Andrews, 1913) and continues through the Kimmeridgian-Tithonian from the oldest *Machimosaurus* species (*M. buffetauti*—lower Kimmeridgian) to the youngest (*M. hugii*—Kimmeridgian to lower Tithonian) (Table 1) (Martin & Vincent, 2013; Young et al., 2014a; Young et al., 2015a). Based on the figures reported in Table 1, DORCM G.3939 is more compatible with *‘Steneosaurus’ obtusidens* than other macrophagous teleosaurids. The late Oxfordian *Machimosaurus* sp. (2007.0.14) does not preserve the anterior part of the mandibular symphysis, and it is then difficult to compare with DORCM G.3939 (Table 1). However, we can estimate ∼24 dentary teeth for Musée de la Princerie 2007.0.14, based on the symphyseal tooth count relative to the splenial in *M. buffetauti* (SMNS 91315), the *Machimosaurus* specimens with the highest tooth count in the genus. This estimate is comparatively lower than our estimate for DORCM G.3939.

Second, the limited lateral extent of the splenial also supports similarity with *‘Steneosaurus’ obtusidens*. As mentioned above, the DORCM G.3939 mandible broke in line with the D19–D20 alveoli, corresponding to an anterior section of the splenial. It follows that the DORCM G.3939 mandibular symphysis was longer than that of *M. buffetauti* (20–21 alveoli) and more similar to *‘Steneosaurus’ obtusidens* (24–25 alveoli) (Andrews, 1913; Martin & Vincent, 2013; Young et al., 2014a; Young et al., 2015a).

Finally, additional evidence supporting close affinity between DORCM G.3939 and *‘Steneosaurus’ obtusidens* comes from the structure of the lower jaw in dorsal view. The posterior end of the teleosaurid mandibular symphysis is marked by a noticeable lateral enlargement of the mandible, due to the divergence of the mandibular rami. This structure can be seen in all teleosaurid specimens (Andrews, 1913; Lepage et al., 2008; Martin & Vincent, 2013; Young et al., 2014a). Critically, such enlargement is not visible in DORCM G.3939. There are two possible explanations for the absence of this feature: (a) such enlargement is absent in DORCM G.3939, or (b) DORCM G.3939 broke anteriorly to the enlargement and therefore the preserved fossil does not show it. We consider explanation (a) unlikely as a lateral enlargement of the rami is consistently shown in all teleosaurids, including well preserved, three-dimensional specimens. The absence of the coronoid and the position of the break along the splenials are compatible with explanation (b). If correct, this corroborates that the symphyseal tooth count in DORCM G.3939 was higher than 19–20 (∼28 estimated dentary teeth), and thus compatible with a ‘long-snouted’ blunt-toothed teleosaurid: *‘Steneosaurus’ obtusidens*.

Multiple lines of evidence demonstrate that DORCM G.3939 is more similar to *‘Steneosaurus’ obtusidens* than it is to *M. buffetauti*. However, we cannot currently recognize any unequivocal synapomorphies that link DORCM G.3939 to ‘*S*.’ *obtusidens*, mostly because the genus *Steneosaurus* is in need of revision. In the absence of unambiguous diagnostic features we conclude that DORCM G.3939 should be provisionally referred to *‘Steneosaurus’* cf. *obtusidens*. Further study of the *‘Steneosaurus’ obtusidens* holotype and new discoveries from the Corallian Gap are needed to confirm this assignment.

Interestingly, the Corallian Nothe Grit Member was deposited under near-shore tidal conditions. The discovery of a macrophagous teleosaurid within such an environmental setting is not surprising, but the absence of piscivorous teleosaurids is. Among teleosaurids, only the macrophagous *Machimosaurus* is hypothesised to have been able to venture into open seas (Young et al., 2014a). Conversely, excluding rare exceptions, piscivorous teleosaurids have mainly been found in lagoonal-coastal environments. The paucity of teleosaurids in the KCF of the UK may be environmental, as this unit was deposited in deeper water than the OCF (Gallois, 2004). It is, however, surprising that the only teleosaurid material within the ‘Corallian Gap’ of the UK is currently represented by macrophagous teleosaurid teeth. A possible explanation may be that, despite similar water depth conditions, the Oxfordian environment would have been very different from the nutrient rich, shallow and warm Callovian seas. However, there is another possibility. The Corallian is less exposed and sampled compared to the industrially-exploited OCF and KCF. The rarity of marine reptile material may simply be a consequence of sampling bias. Support for this statement comes from the report of abundant crocodilian teeth from Smallmouth Sands (lowermost Kimmeridgian) (Young & Steel, 2014), the shallowest deposition depth of the KCF Dorset Succession. It is then possible that teleosaurids became rare in the UK only during the Kimmeridgian, when the British Jurassic seaway transitioned from a coastal to a deep outer-shelf environment (Gallois, 2004). We predict that further studies on the poorly known material from the Corallian Gap will elucidate the time and mode of decline of Teleosauridae in the British Late Jurassic formations.

## Conclusion

In this paper we described the first mandibular material of Teleosaurid from the Corallian Gap of the UK. The specimen can be referred to a sub-clade of macrophagous teleosaurids including *‘Steneosaurus’ obtusidens* and *Machimosaurus* based on its possession of unique craniodental characters. However, there are no clear synapomorphies linking it to another member of this sub-clade, or autapomorphies showing that it is a new taxon. Nevertheless, we showed that the mandible geometry and the tooth count (absolute and relative to the splenial) match those of *‘Steneosaurus’ obtusidens* more closely than any *Machimosaurus* specimens. In absence of unambiguous autapomorphies of *‘Steneosaurus’ obtusidens* or *Machimosaurus buffetauti* we refer DORCM G.3939 to *‘Steneosaurus’* cf. *obtusidens*. Future discoveries of more complete Corallian specimens and the revision of the *‘Steneosaurus’ obtusidens* holotype will clarify if the tooth counts of DORCM G.3939 relative to splenial are indeed compatible with the Callovian teleosaurid. In a broader context, DORCM G.3939 demonstrates that the *‘Steneosaurus’ obtusidens* + *Machimosaurus* lineage was still present in the British formations at the beginning of the Oxfordian.

## Institutional Abbreviations

CAMSM: Sedgwick Museum, Cambridge, United Kingdom
DFMMh: Dinosaurier-Freilichtmuseum Münchehagen, Lower Saxony, Germany
DORCM: Dorset County Museum, Dorchester, United Kingdom
MPV: Musée paléontologique (Paléospace) de Villers-sur-Mer, Normandy
NHMUK: Natural History Museum, London, United Kingdom
NMS: Naturmuseum Solothurn, Switzerland
OUMNH: Oxford University Museum of Natural History, United Kingdom
PETMG: Peterborough Museum & Art Gallery, Peterborough, United Kingdom
SMNS: Staatliches Museum für Naturkunde Stuttgart, Germany

## Additional Information and Declarations

### Data Availability

The following information was supplied regarding data availability: The research in this article did not generate any raw data.
